# Current treatment of IgA nephropathy

**DOI:** 10.1007/s00281-021-00888-3

**Published:** 2021-09-08

**Authors:** Jürgen Floege, Thomas Rauen, Sydney C. W. Tang

**Affiliations:** 1 Division of Nephrology and Rheumatology, RWTH Aachen University Hospital, Pauwelsstr. 30, 52074 Aachen, Germany; 2 Division of Nephrology, Department of Medicine, The University of Hong Kong, Queen Mary Hospital, Pok Fu Lam, Hong Kong

**Keywords:** IgA nephropathy, Mesangioproliferative glomerulonephritis, Supportive therapy, Glucocorticoids, Complement

## Abstract

IgA nephropathy (IgAN) is the most common type of glomerulonephritis in Asia and the Western world. In most patients, it follows an asymptomatic to oligosymptomatic course and GFR loss, if any, is slow. The mainstay of therapy therefore is optimized supportive care, i.e., measures that lower blood pressure, reduce proteinuria, minimize lifestyle risk factors, and otherwise help to reduce non-specific insults to the kidneys. The value of immunosuppression has become controversial and if at all, systemic high-dose corticosteroid therapy should be considered for a few months taking into account patient characteristics that would caution against or preclude such therapy. In addition, adverse events related to corticosteroid therapy markedly increase as GFR declines. Beyond corticosteroids, there is little evidence that any additional immunosuppression is helpful, with the exception of mycophenolate mofetil in patients of Asian descent. A considerable number of clinical trials ranging from enteric coated budesonide to blockade of B-cell function to complement inhibitors are currently ongoing and will hopefully allow a more targeted therapy of high-risk patients with progressive IgAN in the future.

## Introduction

Although IgA nephropathy (IgAN) is the most common type of primary glomerulonephritis (GN) worldwide and has been first described more than 50 years ago, our understanding of the disease pathophysiology is still incomplete and treatment has remained largely empiric [1]. One of the fundamental issues in approaching the treatment of IgAN is that there are few if any animal models that mimic the human disease, in particular its early stages, which severely hampers preclinical trials of new therapeutic agents (refers to the article of Monteiro and Suzuki in this Special Issue).

Clinically, IgAN can follow highly diverse courses ranging from asymptomatic urinary abnormalities with the potential for spontaneous resolution to rapidly progressive GN (RPGN) with kidney failure. In the common manifestation of macroscopic hematuria often in association with an upper respiratory tract infection, recent studies have shown its relationship with acute kidney injury (AKI) where kidney function in up to 25% of patients does not return to baseline [2]. Immediate management should focus on supportive care for AKI and a repeat kidney biopsy be considered to exclude potentially reversible causes if there is no sign of recovery within 2 weeks of resolution of macroscopic hematuria. Hematuria-associated AKI alone is not an indication for immunosuppression. In the very rare patient with a rapidly progressive course and necrotic and crescentic lesions affecting most glomeruli, we only have case series that have yielded conflicting results, namely, some groups advocating immunosuppression with cyclophosphamide and steroids analogous to vasculitis-associated RPGN [3] whereas in other series the renal outcome was dismal with and without immunosuppression [4]. Nevertheless, the revised KDIGO guidelines (www.kdigo.org) suggest immunosuppression in such cases, albeit at a very low level of evidence and confidence. In another very rare situation, i.e., IgAN associated with nephrotic syndrome (i.e., not just nephrotic range proteinuria), there is often a coincidence of minimal change nephropathy and IgA deposits and the KDIGO guidelines suggest treatment analogous to adults with minimal change nephropathy.

The vast majority of adult IgAN patients coming to medical attention are those with a slowly progressive course, often with already significant GFR reduction at first presentation, mild to moderate proteinuria, persistent microhematuria, and hypertension. In the present review, we will focus on this patient group, since randomized controlled trial (RCT) data are available for them and new therapeutic approaches can be expected in the next years.

## What constitutes optimal supportive care?

**Fig. 1:**
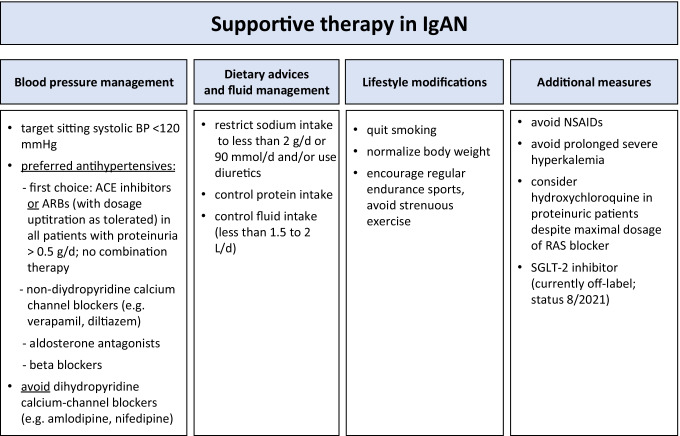
Cornerstones of supportive therapy in IgA nephropathy adapted from [22,23]

Although IgAN bears a wide range of kidney pathologies and clinical courses may be highly variable, the common therapeutic aim in all IgAN patients is to retard disease progression and further decline of kidney function. Since non-specific modifiers such as uncontrolled hypertension and proteinuria potently impact the disease course, it is indisputable that supportive measures targeting either of these processes should first be initialized in all IgAN patients at risk for progressive disease (Fig. 1). It is well-described that blood pressure (BP) increases at very early stages in IgAN patients with a significant activation of the renin-angiotensin system (RAS) in kidneys of these patients. Even patients, who are apparently normotensive, have a higher BP than matched healthy individuals and exhibit subtle cardiac changes [5, 6]. The best evidence available is for blockade of the renin-angiotensin system (RAS) using either ACE inhibitors or angiotensin receptor blockers (ARB) that should be initiated as first-line antihypertensives in all IgAN patients exhibiting a proteinuria above 0.5 g/day, irrespective of whether they are hypertensive or not. This approach is strongly supported by a level 1B recommendation in the revised KDIGO guidelines (www.kdigo.org). Indeed, BP control should primarily be performed using RAS blockers since retrospective registry data showed that IgAN patients treated with an ACE inhibitor to control BP had a better preservation of kidney function than IgAN patients that were not treated with ACE inhibitors or ARBs [7]. Even if BP is well-controlled (i.e., systolic values < 120 mmHg in adult patients), RAS blockers should be uptitrated to the maximum tolerated dose aiming at further reduction of proteinuria. In contrast to RAS blockers, dihydropyridine calcium-channel blockers may not be the best first-line agents to control blood pressure in IgAN, as they induce preglomerular vasodilation and thus are more likely to transmit high blood pressure into the glomerulus [8].

Despite the large body of evidence that supports the use of RAS inhibitors in the vast majority of IgAN patients, there are some uncertainties which have not been addressed in the existing clinical trials. This comprises the question whether RAS blockade is beneficial in normotensive IgAN patients with only moderately increased proteinuria (i.e., around 0.5 g/day). Furthermore, it is not clear whether dual RAS blockade using the combination of an ACE inhibitor and an ARB exerts similar positive effects in IgAN patients as observed in other glomerular diseases. About 20 years ago, smaller clinical trials had demonstrated additional antiproteinuric effects through a co-administration of losartan with ACE inhibitors in IgAN patients [9,10]. However, recent findings from our STOP-IgAN cohort argue against such a dual RAS blocker regimen. Unexpectedly, proteinuria at the end of the randomized, 3-year trial phase was even higher in our patients on dual RAS blocker therapy whereas overall renal outcomes were comparable between trial participants under single and those dual RAS blocker therapy [11]. Another RAS-blocking therapy, the direct renin inhibitor aliskiren, has been shown to reduce proteinuria at 6 months by a further 26% and suppress serum IL-6 and TGF-β levels when given to IgAN patients with proteinuria > 1 g/day despite optimized ARB treatment [12]. The antiproteinuric effect was reproduced in another small, randomized cross-over study [13]. Nevertheless, aliskiren was not pursued further due to a high incidence of hyperkalemia and hypotension among type 2 diabetics with CKD.

Another emerging supportive approach in patients with high-risk IgAN is the addition of a sodium-glucose cotransporter-2 (SGLT2) inhibitor (Fig. 1). In the DAPA-CKD trial, 270 IgAN patients (mostly without concomitant diabetes) with a low median eGFR around 40 ml/min received dapagliflozin on top of a RAS inhibitor [14]. Renal outcome markedly improved and the hazard ratio for the renal endpoint (50% loss of eGFR, dialysis, or death from a kidney disease-related or cardiovascular cause) was 0.29 (95% confidence interval, 0.12, 0.73) compared to placebo [15]. Limitations of that study were its post hoc nature, relatively few patients reaching renal endpoints, and in particular an unusually bad outcome of the placebo group [16]. More insight into the role of SGLT2 inhibitors in the treatment of IgAN will be provided by the ongoing EMPA-Kidney trial, in which non-diabetic patients, many with underlying IgAN, are randomized to empagliflozin or placebo on top of a RAS inhibitor (ClinicalTrials.gov Identifier: NCT03594110).

Beyond antihypertensive and antiproteinuric medications, intense patient lifestyle education on diet, physical activity, body weight reduction, smoking cessation, analgesic use, and other factors should be integral part of the supportive therapy. Apart from dietary sodium restriction to an intake below 2 g/day (< 90 mmol/day), no additional dietary interventions have proven to affect outcomes in IgAN patients. Several, but not all studies suggested that obesity accelerates disease progression in IgAN patients [17,18,19

In our practical experience, at least 6 months are required to institute and/or optimize all supportive care measures (Fig. 1) even in patients who are not naïve to therapy. Other approaches, in particular immunosuppression, should not be considered until all such measures are in place.

## What is the role of corticosteroid therapy?

In the absence of a true RPGN course of IgAN, i.e., not isolated crescents in the kidney biopsy and no rapid GFR decline, supportive therapy remains the mainstay of treatment for adults with IgAN. Optimization of such therapy (see preceding chapter) requires time and usually cannot be achieved in a few weeks even in patients already receiving RAS blockade. Thus, the question of immunosuppression, in particular corticosteroid therapy, should not be posed too quickly. This is best evidenced by our STOP-IgAN trial, where simply the optimization of supportive care resulted in a substantial proportion of patients dropping proteinuria to less than 1 or 0.75 g/day [21], which is usually the threshold considered for immunosuppression or enrollment into clinical trials.

Prior to 2010, three RCTs had shown that a 6-month course of systemic corticosteroids given to proteinuric IgAN patients with a GFR about 50 ml/min reduced both proteinuria and the risk of progressive kidney failure [22–25]. Treatment efficacy was independent of whether the therapy consisted of combined pulse and oral steroids or an exclusively oral regimen. However, a smaller North American RCT using alternating corticosteroid therapy (prednisone 60 mg/m^2^ every other day tapered to 30 mg/m^2^ until the end of year 2) noted no benefit for kidney outcomes [26] as did a Japanese trial using a low-dose regimen (20 mg prednisone per day tapered over 2 years) [27]. Successful corticosteroid re-treatments in patients whose proteinuria relapsed following prior steroid therapy has been reported [28]. The notable features of all of these early trials are the reported relative absence of serious adverse events even with the more aggressive corticosteroid regimens and the fact that RAS blockade was not used consistently or at maximum tolerated doses. Also, none of the trials mentioned any other supportive measures.

**Fig. 2:**
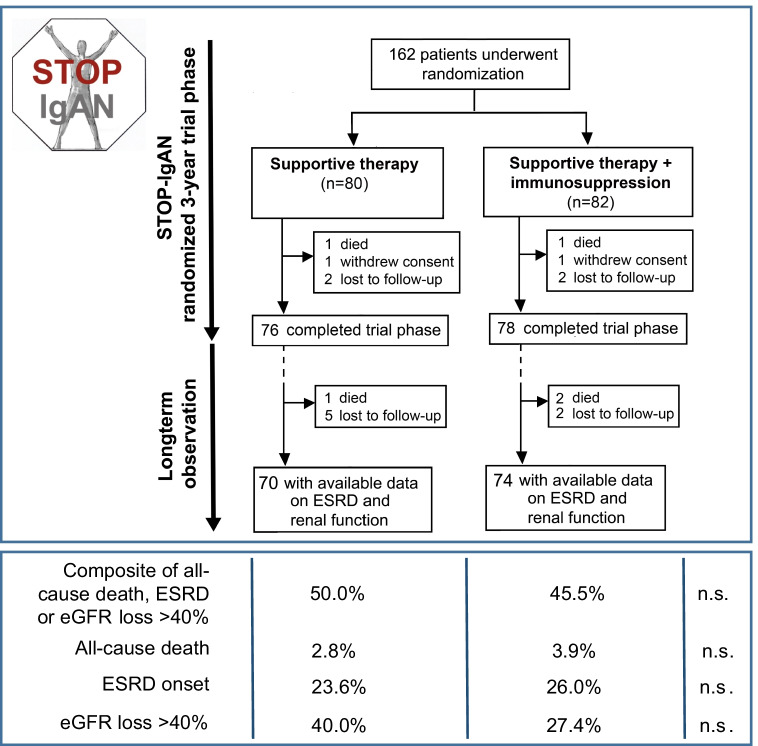
Patients and key study results from the long-term observation of the STOP-IgAN cohort [24,32]

Since 2010, two major RCTs on corticosteroids in high-risk patients have been published [21, 29, 30]. In the STOP-IgAN trial, we first optimized supportive care, in particular RAS blockade for 6 months. Only if patients continued to have a proteinuria more than 0.75 g/day and if they had an eGFR above 60 ml/min, they were randomized to continue on supportive care only or to receive additional combined pulse and oral corticosteroids. Neither at the end of the trial, i.e., 3 years after randomization, nor at prolonged follow-up of a median of more than 7 years was there a detectable benefit for renal outcomes [21, 29]. The high risk of the patient cohort was evidenced by the fact that at 10 years almost 70% had reached a combined endpoint of death, dialysis, or 40% loss of eGFR, clearly demonstrating that more effective and different therapy is needed for such patients [21]. Adverse events, in particular infections, almost doubled in the corticosteroid-treated patients and there were considerable increases in the patients reporting weight gain above 5 kg and the induction of glucose intolerance or diabetes [31]. The second large trial is the TESTING RCT [30]. In this trial, high-risk Asian patients were given a RAS blocker for at least 3 months and then randomized to placebo or oral methylprednisolone (0.6–0.8 mg/kg/day; max 48 mg/day and tapered over 6–8 months). Patients in TESTING had a mean proteinuria of 2.4 g/day, notably higher than the patients enrolled in our STOP-IgAN trial (1.6–1.8 g of proteinuria per day), whereas average eGFR at baseline was similar and around 60 ml/min. TESTING had to be halted after randomization of 262 subjects, given an 11% greater risk of serious adverse events in the methylprednisolone arm, including two deaths related to infectious complications [30]. Even with this premature stop, the primary kidney outcome (composite of 40% eGFR reduction, dialysis, and death due to kidney disease) occurred less frequently in the methylprednisolone arm. Of note, in TESTING patients in the placebo arm exhibited an annual eGFR loss of about 7 ml/min/1.73 m^2^ [30], notably higher than in our STOP-IgAN trial where the annual eGFR loss was only 1.5 ml/min/1.73 m^2^ in the control arm [21]. Whether this relates to racial differences, selection of different patients, or differences in the extent of supportive care remains speculative. A follow-up study (TESTING low dose; NCT01560052) with a 50% reduced methylprednisolone dose is currently underway (Fig. 2).

From the above it is clear that, in contrast to earlier reports, there is a significant risk of toxicity related to high-dose corticosteroid therapy in IgAN patients and that there are controversial findings on the efficacy of such therapy. The KDIGO guidelines therefore note in a practice point that “*Clinical benefit of corticosteroids in IgAN is not established and should be given with extreme caution or avoided entirely in the situations*” shown in Table 1 (REF).

**Table 1:** Situations in which corticosteroids should be used with extreme caution or avoided entirely in patients with IgAN (modified from KDIGO 2021 CLINICAL PRACTICE GUIDELINE FOR THEMANAGEMENT OF GLOMERULAR DISEASES. Kidney Int Suppl 2021 in press.)

•eGFR < 30 ml/min/1.73 m^2^.
•Diabetes.
•Obesity (body mass index > 30 kg/m^2^).
•Latent infections (e.g., viral hepatitis, tuberculosis).
•Secondary IgAN (e.g., liver cirrhosis).
•Acute peptic ulceration.
•Uncontrolled psychiatric illness.
•Severe osteoporosis.

## Immunosuppression beyond corticosteroids (Table 2)

**Table 2:** Immunosuppression beyond corticosteroids in IgAN ( modified from KDIGO 2021 CLINICAL PRACTICE GUIDELINE FOR THEMANAGEMENT OF GLOMERULAR DISEASES. Kidney Int Suppl 2021 in press.)

Agent	Suggested usage
Azathioprine	Not recommended
Cyclophosphamide	Not recommended unless in the setting of rapidly progressive IgAN
Calcineurin inhibitors	Not recommended
Rituximab	Not recommended
Mycophenolate mofetil	Chinese—can be considered as a steroid-sparing agent Non-Chinese—insufficient evidence for efficacy
Hydroxychloroquine	Chinese—in patients at high risk of progression despite optimized supportive care Non-Chinese—no data available

As the putative pathologic polymeric IgA in IgAN is derived from a specific subset of B cells, there has been considerable interest in depleting B cells as a therapeutic approach. In an open label, multicenter study [32], 34 adult patients with biopsy-proven IgAN, persistent proteinuria > 1 g/day while maintained on a RAS blocker were randomized to receive standard therapy or with the addition of rituximab. After 1 year, rituximab therapy did not significantly improve kidney function or proteinuria nor did it reduce serum levels of galactose-deficient IgA1 and antigalactose-deficient IgA1 antibodies. Therefore, anti-CD20 therapy is currently not a recommended treatment for IgAN. Likewise, there is insufficient evidence to show its efficacy in IgAV although in a small series of 12 patients aged 19–75 years with IgAV and crescentic nephritis, 11 achieved a clinical response at 6 months [33]. In another small cohort study, 20 of 22 patients who received rituximab for refractory or relapsing IgAV achieved remission and of these 7 had a subsequent relapse [34].

MMF, being a B-lympholytic agent, has also been studied in IgAN with 6 RCTs in various populations published. Although these trials have produced conflicting results, they differ significantly in quality, patient selection, and treatment duration. Three studies conducted in Chinese patients have shown a beneficial effect of MMF. The first trial from Beijing included 62 patients with severe IgAN and proteinuria of > 2 g/day. Patients who received MMF showed significant improvement in proteinuria and serum lipid levels compared with those who received prednisone [35]. The second study from Hong Kong included 40 patients with mild tubulointerstitial lesions and persistent proteinuria of > 1 g/day despite RAS blockade [36]. Mycophenolate mofetil treatment for 6 months resulted in significant reduction in proteinuria and improved kidney survival at 6-year follow-up compared with using RAS blocker alone [37]. The last is a multicenter RCT in which 6 months of MMF plus low-dose corticosteroid was non-inferior to standard-dose corticosteroid treatment among 174 incident IgAN patients presenting with proliferative lesions (E or C lesions with or without necrosis) and proteinuria > 1 g/day [38]. Steroid-related adverse effects were much lower in the MMF group. Another three studies conducted in Caucasian patients showed mixed results. The first included 34 Belgian patients with impaired kidney function, histologically unfavorable criteria, and arterial hypertension [39]. All patients received salt restriction and ACE inhibitor therapy, and high-dose MMF (3 g/day) treatment for 3 years failed to demonstrate a beneficial effect. In a similar study performed in the USA that recruited patients with even more advanced renal insufficiency using MMF at 2 g/day as a “salvage” therapy, a worse outcome was observed in the MMF group [40]. The last RCT, conducted in 52 children, adolescents, and adults from the USA and Canada with persistent proteinuria (UACR ≥ 0.6 g/g in males or 0.8 g/g in females) despite RAS blockade and fish oil treatment for 3 months, was terminated early as MMF did not reduce proteinuria at 6 and 12 months after treatment initiation [38]. Given these mixed results across different ethnic groups and given that none of these studies was adequately powered to provide a definitive answer, the KDIGO guidelines therefore note in a practice point that MMF can be considered as a steroid-sparing agent only in Chinese patients in whom corticosteroids are being considered.

Cyclophosphamide is not commonly used for IgAN in patients with a chronic indolent clinical course. It is only indicated in a subgroup of patients at risk of progressive loss of kidney function, namely, those with crescentic glomerular lesions and rapidly progressive clinical course [41, 42]. Despite limited evidence, the KDIGO guidelines also included a practice point for using cyclophosphamide in children with rapidly progressive IgAN [43].

Calcineurin inhibitors have been studied since a long time ago and have not been featured as a recommended treatment for IgAN. An earlier study on cyclosporine showed lack of efficacy and nephrotoxicity [44]. A more recent meta-analysis of 10 relevant studies involving 472 patients showed that tacrolimus combined with corticosteroid significantly reduced proteinuria compared with control patients without increasing adverse events [45]. Its long-term efficacy and safety in IgAN remains to be tested.

An early retrospective analysis of 74 patients followed for 10 years showed that long-term azathioprine combined with low-dose prednisone did not alter the clinical course compared to untreated controls [46]. A more recent prospective randomized study of 207 subjects showed that the addition of azathioprine to corticosteroids did not confer additional benefits in terms of kidney survival versus corticosteroids alone in patients with proteinuria ≥ 1 g/day and serum creatinine ≤ 2.0 mg/dl [28]. Available data therefore suggest that azathioprine is ineffective and may even be toxic in IgAN.

Hydroxychloroquine (HCQ) is an antimalarial agent that is widely used in patients with rheumatoid arthritis and systemic lupus erythematosus. From a pathophysiological point of view, beneficial effects of HCQ may relate to its antagonism to the Toll-like receptor (TLR)-9, which has been shown to be upregulated in the pathogenesis of experimental IgAN-like mice models [47, 48]. The use of HCQ in IgAN patients arose from a few case–control studies in Chinese IgAN patients [49, 50]. In a small, short-term RCT of 60 subjects from China, HCQ was given to patients with proteinuria of 0.75–3.5 g/day despite optimized RAS blockade reduced proteinuria by 48% versus 10% in the placebo group at 6 months [51]. There were no severe adverse effects reported in the HCQ group. A larger RCT from the same center is currently underway (NCT02765594). The KDIGO guidelines provide in a practice point that HCQ can be considered in Chinese patients who remain at high risk of progression despite optimized supportive care (Fig. 1).

## Are there suitable biomarkers to guide therapy?

The KDIGO guidelines categorically state in a practice point that to date there are no validated diagnostic serum or urine biomarkers for IgAN. Nevertheless, over the years, researchers have been keen in searching these markers for diagnosis and for introducing a more personalized therapy for IgAN.

### Serum biomarkers

Galactose-deficient (Gd) IgA1 and the corresponding autoantibodies that recognize it are the most studied serum biomarkers [52, 53]. High circulating levels of Gd-IgA1 have been observed in all individuals from a US cohort of familial patients with IgAN, in 47% of their at-risk relatives, but only 5% of unrelated individuals who married into the family [54]. Similar findings were detected in Chinese patients [55] in whom patients with familial IgAN had higher serum Gd-IgA1 levels than those with sporadic disease. Polymeric IgA1 isolated from familial clusters showed enhanced binding to mesangial cells with increased release of inflammatory cytokines. Serum Gd-IgA1 levels from patients with sporadic or familial IgAN and relatives of those with familial IgAN were higher than those of healthy controls.

Quantification of serum levels of Gd-IgA1 as a diagnostic test has been developed [56, 57]. ROC analyses revealed 77% sensitivity and 90% specificity for serum Gd-IgA1 to distinguish IgAN patients from healthy controls. In addition, serum levels of IgA, Gd-IgA1-specific IgG, and Gd-IgA1-specific IgA were shown to be elevated in patients with IgAN versus healthy controls and patients with other kidney diseases [58]. Among them, Gd-IgA1-specific IgG showed the best performance for diagnosing IgAN, with 89% sensitivity and 92% specificity. Although these biomarkers may be potentially useful for the diagnosis of IgAN, there is substantial overlap in serum levels of individual biomarkers between patients with IgAN, other kidney diseases, and healthy controls. Thus, no single biomarker was sufficiently specific for IgAN, suggesting that a panel of serum biomarkers may be required to confidently differentiate IgAN from other glomerular diseases [59]. A Korean study observed a significant decrease of Gd-IgA1 from 0 to 3 months of immunosuppressive therapy in kidney transplant recipients with IgAN and concluded that prednisone may influence serum levels of Gd-IgA1 [60].

Another potential serum biomarker may be miRNAs that regulate gene expression. In a retrospective international study of biopsy-proven IgAN patients from Hong Kong, Japan, Greece, and Italy, the combined biomarkers of circulating miR-148b and let-7b were found to discriminate patients with primary IgAN from patients affected by other forms of primary glomerulonephritis with an area under the receiver operating characteristic curve of 0.76 [61].

### Urine biomarkers

Proteinuria is a time-honored biomarker for monitoring the progression of kidney damage [62], but this biomarker is not specific for IgAN. A fraction of Gd-IgA1 from the glomerular deposits is excreted into the urine and thus could potentially represent a disease-specific marker of IgAN. In a study of 207 patients with IgAN, 205 patients with other kidney diseases, and 57 healthy controls recruited in USA, Japan, and Italy, urinary excretion of Gd-IgA1 discriminated patients with IgAN from patients with other proteinuric kidney diseases [63]. Furthermore, urinary Gd-IgA1 levels showed a good correlation with proteinuria in patients with IgAN. The utility of urinary Gd-IgA1 is further supported by its specific detection in the glomeruli of IgAN and IgAVN patients but not in patients with other kidney diseases including lupus nephritis, HCV-related nephropathy, and membranous nephropathy [64].

Podocyte depletion causes glomerular sclerosis and its loss in urine is another potential marker of disease progression. Urinary podocyte messenger RNA may integrate the prognostic value of proteinuria for decision-making in therapy [65].

Increased urinary levels of inflammatory cytokines and growth factors are observed in patients with IgAN [66]. Indeed, increased levels of urinary cytokines and/or growth factors might be associated with advanced histopathologic changes but provide little diagnostic value. Whether they can be used for guiding therapy can be an area of research. Urinary peptides could be of future interest for developing diagnostic and prognostic biomarkers that are relevant to IgAN. Such markers may be developed, for example, using urinary peptidomics [67, 68].

### Should MEST-C and crescents influence treatment?

The KDIGO guidelines specifically point out in a practice point that there are no validated prognostic serum or urine biomarkers for IgAN other than eGFR and proteinuria. Except for urinary peptidomics that has a potential in the future, the serum and urine biomarkers stated above cannot be used to guide therapy. The International IgAN Prediction Tool [69] that takes into account baseline clinical and demographic characteristics and treatment received at the time of biopsy and the MEST-C score [70] is a valuable resource to quantify the risk of progression and inform shared decision-making with patients. However, it cannot be used to determine the likely impact of any particular treatment regimen. Of note, the tool is derived from large cohorts of adults with biopsy-proven IgAN from Europe, North America, China, and Japan.

The KDIGO guidelines also state in a practice point that there is insufficient evidence to support the use of the Oxford Classification MEST-C score in determining whether immunosuppression should be commenced in IgAN and to base treatment decisions on the presence and number of crescents in the kidney biopsy. Histologic grading and crescents therefore cannot be used to influence therapy. It is important to note that the mere presence of crescents on a kidney biopsy in a patient with otherwise stable kidney function is not an indication for initiating immunosuppressive therapy, which has to take into account the overall clinical presentation to determine whether it resembles rapidly progressive IgAN.

## Synopsis algorithm

**Fig. 3:**
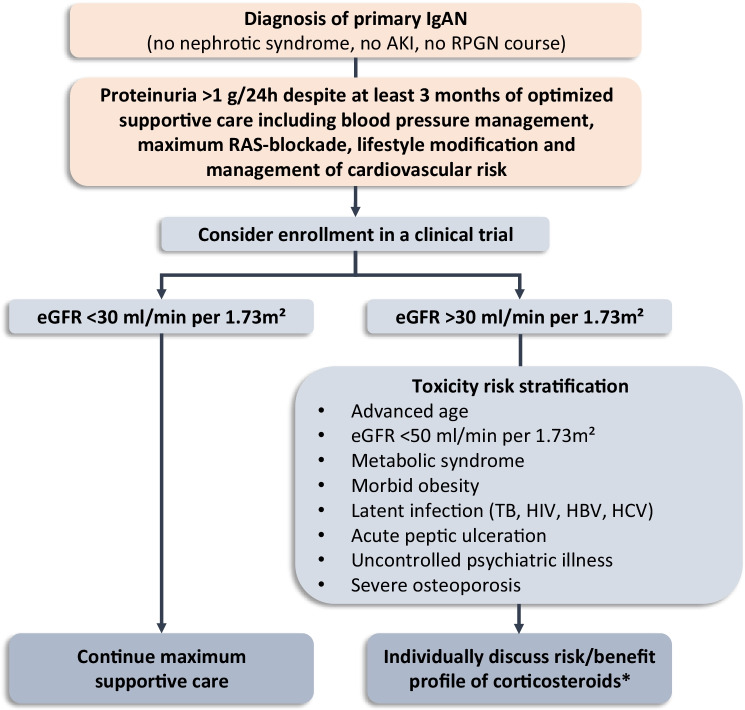
Treatment of primary IgAN (adapted from KDIGO 2021 CLINICAL PRACTICE GUIDELINE FOR THE MANAGEMENT OF GLOMERULAR DISEASES. Kidney Int Suppl 2021 in press.). *Specific considerations apply to some ethnic groups: (1) if Japanese, consider tonsillectomy; (2) if Chinese consider mycophenolate mofetil as a corticosteroid-sparing agent

A synopsis algorithm of how to treat patients with primary IgAN, lacking unusual features (i.e., without AKI, nephrotic syndrome, or a rapidly progressive GFR loss), is shown in Fig. 3.

## Emerging treatment approaches and ongoing trials

All recent trials stress that we have a high unmet medical need for more efficacious and safer therapies in IgAN patients at high risk for progressive kidney disease. It is thus very gratifying to see that after many years of relative neglect, a number of pharmaceutical companies have finally embarked on trials in such patients. A central reason that has boosted the number of RCTs in IgAN is the acceptance of regulatory bodies of surrogate outcome parameters as endpoints in such trials, namely, reduction in proteinuria and the decline of the slope of annual eGFR measurements [71, 72].

At the time of writing this article, several phase III RCTs are still ongoing in IgAN. Essentially all trials include patients with an eGFR above 30 ml/min, proteinuria usually above 1 g/day and only after RAS blockade had been optimized for several weeks or months:

1. In the NEFIGARD trial (ClinicalTrials.gov Identifier: NCT03643965), patients are randomized to placebo or enteric release budesonide (Nefecon®) based on a phase II RCT, which demonstrated that this enteric corticosteroid reduced proteinuria and eGFR loss over 1 year in IgAN [73].
2. In the ARTEMIS trial (ClinicalTrials.gov Identifier: NCT03608033), patients are randomized to placebo or repeated infusions of an antibody to MASP-2, the key enzyme regulating the activity of the mannose-binding lectin pathway of complement. In a small phase II RCT, this antibody had markedly reduced proteinuria in IgAN patients [74].
3. There are more phase II or III clinical trials to evaluate the efficacy and safety of inhibiting other arms of the complement cascade in IgAN, for example, (i) alternative pathway—using LNP023, an orally available, small-molecule inhibitor of complement factor B (APPLAUSE trial; ClinicalTrials.gov Identifier: NCT04578834) in which appropriate prior vaccinations against pneumococci, meningococci, and haemophilus influenzae B are needed and (ii) terminal converging pathway—using ravulizumab, a C5 inhibitor (ClinicalTrials.gov Identifier: NCT04564339).
  Yet, another non-immunosuppressive combination therapy approach gained attention in IgAN and other glomerular diseases over the last years, i.e., a new-class drug of a dual acting ARB and endothelin receptor antagonist (ERA) named sparsentan. Preclinical studies in animal models demonstrated that ERAs may reduce proteinuria by ameliorating kidney damage and in subsequent trials a combined ERA/ARB therapy exerted additive antiproteinuric effects in patients with glomerular diseases such as diabetic nephropathy, FSGS, and IgA nephropathy [75–77]. As such, ERAs emerge as a highly promising novel treatment principle that may even augment effects of a single RASB therapy:
4. In the phase III PROTECT trial (ClinicalTrials.gov Identifier: NCT03762850), patients are randomized to receive irbesartan or sparsentan, a dual angiotensin-II and endothelin-1 receptor blocker, based on a phase II trial in patients with focal segmental glomerulosclerosis [77].
5. Similar to PROTECT, another RCT (ALIGN; ClinicalTrials.gov Identifier: NCT04573478) also targets endothelin-1 using the specific endothelin-A receptor blocker atrasentan.

The good news for the IgAN community is that the above phase III trials target fundamentally different processes in IgAN, ranging from supportive care optimization (PROTECT) to conceivably IgAN-specific pathophysiology (NEFIGARD) and complement inhibition, which likely has a role in many kidney diseases (ARTEMIS). Which of these approaches is the best and safest one, and whether ultimately combinations may be useful in high-risk IgAN patients should become clear in the next years.

Beyond the phase II and III trials mentioned above, a number of phase II trials is currently also underway in high-risk IgAN patients (please refer to www.clinicaltrials.gov for detailed information). Targets mostly include factors involved in B-cell maturation (e.g., BAFF or a proliferation inducing ligand (APRIL)) and immune responses (e.g., atacicept).

We are confident that in 5–10 years from now, we have more specific therapy in progressive IgAN that hopefully can provide more benefit at less risk than today’s relatively non-specific treatment approaches. In particular, we look forward to (a) a greater supportive care armamentarium, (b) new options targeting the dysregulated production of undergalactosylated IgA in IgAN, as well as to (c) new options to halt intraglomerular inflammation and scaring, be it via complement inhibition and/or antifibrotic measures. 
